# MRI and CT findings in anterior thoracic myelomeningocele associated with scoliosis: A case of an 8-year-old child

**DOI:** 10.1016/j.radcr.2023.08.058

**Published:** 2023-08-25

**Authors:** Patient Aganze Migabo, Abasi Amisi Bulabula, Roméo Murhega Bujiriri, Marius Baguma, Léon-Emmanuel Mukengeshayi Mubenga, Ghislain Balemba Maheshe

**Affiliations:** a Department of Radiology, Hôpital Provincial Général de Référence de Bukavu (HPGRB), South Kivu, Democratic Republic of the Congo; b Faculty of Medicine, Université Catholique de Bukavu (UCB), South Kivu, Democratic Republic of the Congo; c Neurology Ward, Hôpital Provincial Général de Référence de Bukavu (HPGRB), South Kivu, Democratic Republic of the Congo; d Center for Tropical Diseases and Global Health (CTDGH), Université Catholique de Bukavu (UCB), South Kivu, Democratic Republic of the Congo; e Surgery Department, Hôpital Provincial Général de Référence de Bukavu (HPGRB), South Kivu, Democratic Republic of the Congo

**Keywords:** Myelomeningocele, Anterior thoracic, Magnetic Resonance Imaging, Democratic Republic of the Congo

## Abstract

Anterior Thoracic Myelomeningocele is a very rare condition. The diagnosis may be made before or after birth. Association with loss of physiologic curvature of the spine is common. We report the case of an 8-year-old boy with congenital dorsal sinistro-convex scoliosis, which was considered nonspecific and improved slightly after physical therapy. The onset of urinary and fecal incontinence at the age of 8 years led the parents to consult a urologist. MRI of the spinal cord revealed an anterior thoracic heterogeneous cystic lesion extending from the third to the eighth dorsal vertebrae. The heterogeneous cystic mass contained a solid spinal cord-like structure on all sequences. No abnormal enlargement, no torsion of the cauda equina or cerebellar amygdala. No malformation of the posterior fossa. CT scan showed aplasia of the right pedicle of the third thoracic vertebrae (T3) with thoracic scoliosis. The associated anterior thoracic myelomeningocele was the final diagnosis that motivated the transfer of the patient to a specialized neurosurgical center.

## Introduction

Spinal dysraphism (SD) is a general term for congenital anomalies of the midline of the neural tube. The midline closure of bone, neural, or other mesenchymal tissues is defective [1,2]. These malformations result from defective midline closure of the osseous, mesenchymal, and neural tissues during the second to sixth weeks of gestation [2]. SD is the most common central nervous system malformations, with myelomeningocele accounting for 85% of reported cases [1]. Worldwide, the incidence and prevalence of SD are estimated to be 0.5-2.5 and 1-3 per 1000 live births, respectively, with recognized geographic differences [1,3,4]. The socioeconomic status of patients, along with other factors such as genetics, environment, and nutrition, contributes significantly to the disparity in the worldwide distribution of SD [2,3,5].

Most SD conditions are diagnosed during pregnancy or shortly after birth [3]. However, some others are detected later in life (childhood or adulthood) due to a lack of clinical manifestations or appropriate diagnostic infrastructure (such as medical imaging). As myelomeningocele and other types of SD are compatible with life, their early diagnosis has several advantages, such as counseling parents, planning treatment options, and minimizing potential associated morbidity [1,2,6].

Approximately 90% of all reported myelomeningoceles are located in the lumbosacral region, followed by the thoracic and cervical spine [1]. Less common is the anterior thoracic myelomeningocele [6]. To the best of our knowledge, no case of anterior thoracic myelomeningocele has been reported in Africa.

The aim of this paper is to report a rare case of anterior thoracic myelomeningocele and to highlight the importance of medical imaging in the diagnosis of this condition.

## Case presentation

An 8-year-old boy with a long history of scoliosis and regular physiotherapy at a rehabilitation center was admitted to the urology unit of a university teaching hospital in Bukavu, eastern Democratic Republic of Congo (DRC), for new-onset urinary and fecal incontinence.

The patient had undergone several spinal radiographs over the past 5 years, which showed only isolated scoliosis. The parents did not recall any possible exposure to mining or other ionizing sources before or during pregnancy. A spinal cord MRI was performed for further etiologic investigation of these new neurologic symptoms. A 0.45 Tesla machine from Hitachi (Aperto by Hitachi, India) helped to perform multiplanar sequences such as T1 weighted image-Fast spin echo (T1WI FSE), T2 weighted image-Fast spin echo (T2WI FSE), T2 Short tau inversion recovery (STIR) and T1 fat sat postgadolinium.

**Fig. 1:**
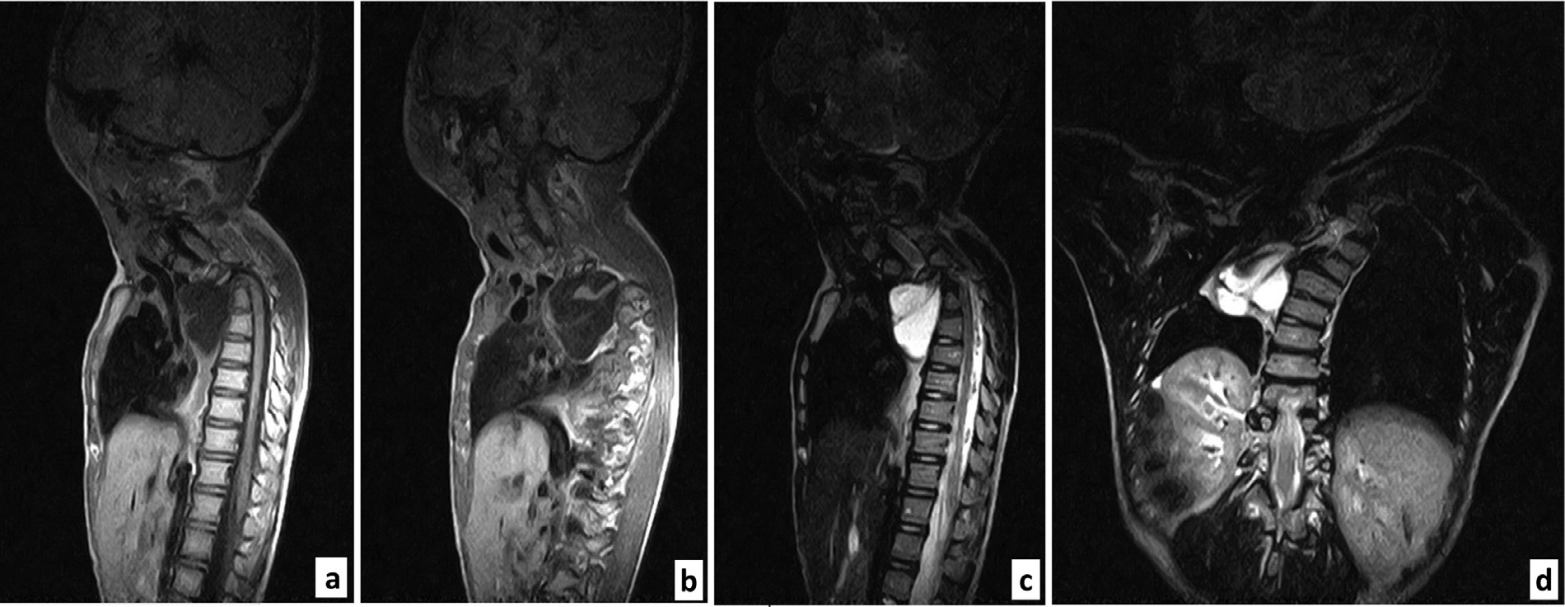
Thoracolumbar spinal MRI. From left to right: Sagittal slices in T1WI Gd(-) through the median plane (A) and the right-side transforaminal plane (B); 2 slices in T2WI, midsagittal (C) and coronal scans (D). Illustration of a grossly rounded prevertebral mass invariably isointense to CSF and that encompassed rare bands mimicking the medullar signal (A, B, C). This right-sided large cyst suggested an extrusion from the spinal canal through T3-T4 right foramen (D).

An anterior right cystic mass (approximately 60.7 × 48.4 × 42.1 mm or 61.84 mL) was visualized at the level of the third (T3) to eighth (T8) thoracic vertebrae. An isointense filiform structure could be seen within the mass, which proved to be the spinal cord. The mass showed an inhomogeneous signal on both T1- and T2-weighted images, globally hypointense on T1 (Figs. 1, A and B), and hyperintense on T2 (Figs. 1, C and D). Postgadolinium sequences showed no patent enhancement. The right neural foramina T3-T4 appeared distended while their bodies were fused (Figs. 1, A–D), contributing to the global appearance of levoscoliosis. The right lung was compressed and the trachea was discreetly pushed anteriorly. Of note, the right hemithorax appeared hypoinflated, possibly due to long-standing passive atelectasis (Fig. 1D).

**Fig. 2:**
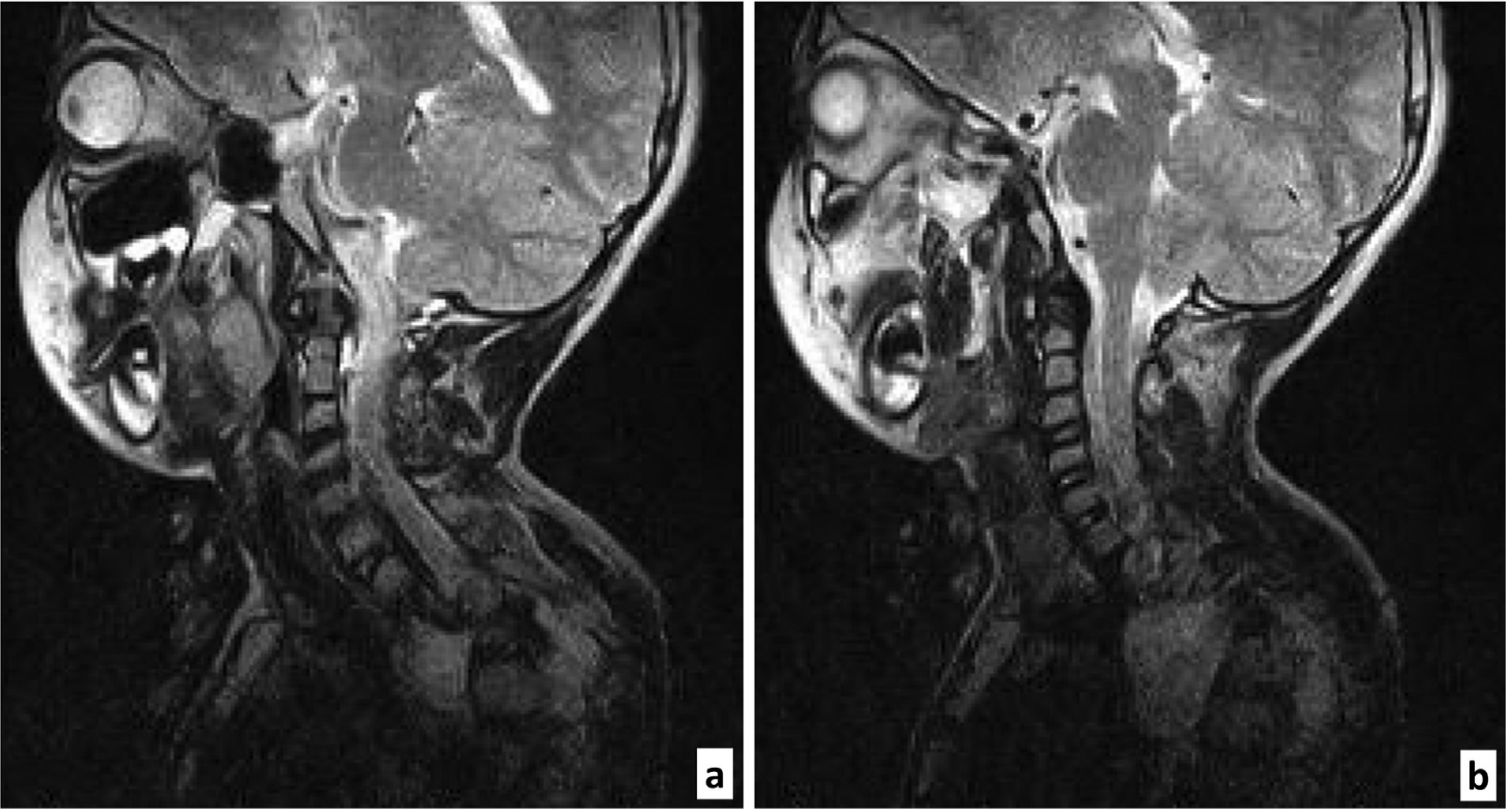
MRI craniocervical junction. Midsagittal (A) and right paramedian (B) slices in T2WI FSE. No engagement seen nor any abnormality of the posterior fossa.

For completeness, 2 sequences centered at the craniocervical junction were subsequently performed to look for possible associated posterior fossa malformations. This study did not reveal any additional lesions (Figs. 2, A and B).

**Fig. 3:**
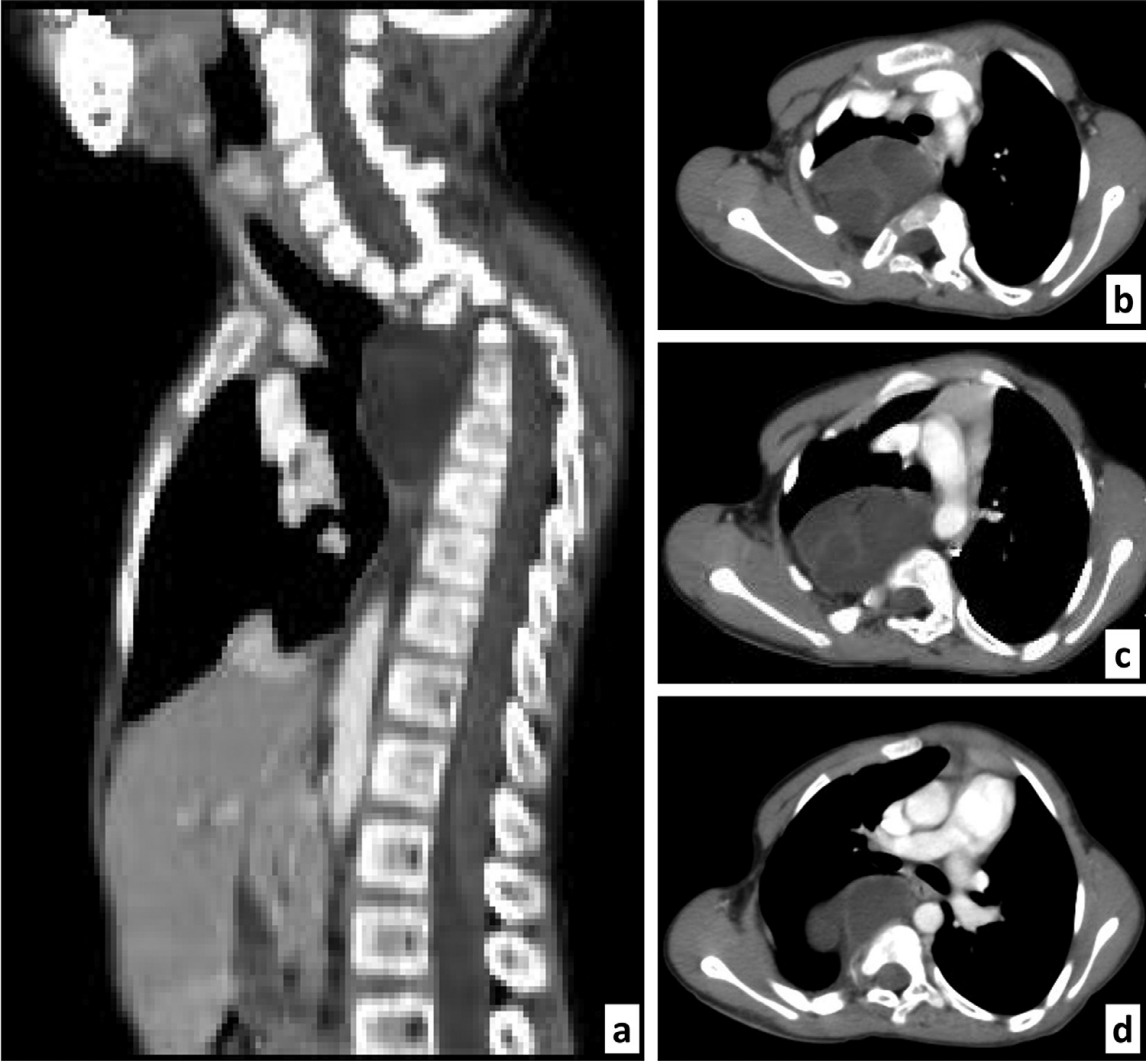
Contrast-enhanced chest CT scan, mediastinal window. Both the midsagittal slice (A) and the 2 axial transpedicular slices at T3 (B,C) and T4 vertebrae (D) demonstrated the T3 right pedicle defect that made a way to an extruded inhomogeneous mass (signals iso- to hypodense to the parietal muscles). Note the right shifted dural sac (B,C,D).

Overall, the diagnosis of isolated anterior thoracic myelomeningocele with levoscoliosis was maintained. There was no obvious pulmonary complication at the time of diagnosis. The patient was referred to a tertiary neurosurgical center for multidisciplinary management. Of note, the findings of the preoperative chest CT scan (64-slice Somatom, Siemens, Germany) performed 2 months later revealed the T3 right pedicle defect (Figs. 3, A–D).

## Discussion

Myelomeningocele is the commonest anatomic subtype of SD, the most recurrent central nervous system malformation, but still compatible with life [1,2,7]. In most African countries, the annual incidence of SD (per 1000 live births) ranges from 0.4 to 1.99 [8]. However, some countries have a much higher incidence, such as Nigeria (about 7) and South Africa (0.77-6.1), while the USA reports 0.2-0.4 [1,9,10]. This geographic variation in prevalence may be closely related to the socioeconomic situation of different countries, which may or may not facilitate the implementation of an effective preventive plan (such as fortified foods) [1]. In fact, folic acid supplementation either in the preconception period or during pregnancy can significantly reduce the incidence of myelomeningocele, as observed in some developed countries such as the United States and Canada [2]. In the Democratic Republic of Congo (DRC), a team of researchers reported on a series of 27 cases of spina bifida treated surgically over an 18-month period. They surveyed nutritional knowledge and practices among women of reproductive age and found only low levels of folate awareness [8].

Low-income countries do not have sufficient and adequate health facilities to screen and diagnose such congenital malformations. Ahuka et al. [5], has shown an increase in congenital malformations, with CNS/neural tube defects topping the list in the eastern part of the DRC. According to the authors, this increase is due to the inaccessibility or destruction of health facilities and to poverty as an indirect and multifactorial consequence of the ongoing civil conflicts in this region.

For anterior cervico-thoracic myelomeningocele, one should distinguish between true and false anterior cases, according to the localization of the defect. False cases, mostly associated with neuroforamen enlargement due to congenital mesenchymal pathology, are well described (more than 100 cases reported) [11]. However, true cases of anterior thoracic myelomeningoceles are rare entities and only a few cases have been reported in the literature [6,11,12]. Depending on its size, it is seen as an enlarged mediastinum or as a rounded parenchymal opacity arising from the mediastinum [1,4,7].

Awareness and early detection of such malformations may prevent neurologic complications in a setting where surgical intervention is possible. In this case report, the presence of a mediastinal mass remained undetected for several years, as all radiographic studies showed only isolated scoliosis, until the onset of neurologic symptoms. This may be explained by either the small size of the mediastinal mass at the time of previous visits, the lack of training in imaging diagnosis, or most likely the under-equipped health centers.

Prenatal screening also includes alpha-fetoprotein dosage and fetal ultrasound [1]. It should be noted that in the case of closed SD, MRI is preferable to ultrasound. The former performs better in assessing the overlying tissues involved in the neural defect, with/without spinal cord involvement [13]. The critical role of neuroimaging cannot be overemphasized. It is important for diagnosis and postoperative evaluation, detection of associated malformations, and treatment planning [1,14]. MRI is not the frontline modality for SD but remains the best morphological modality to study soft tissues, especially the central nervous system [6].

This case report highlights its place in the diagnosis of SDs. Apart from the final diagnosis, imaging modalities with 3D postprocessing allow to establish anatomical relationships in the preoperative evaluation [5]. The history of irrelevant previous radiographs and the new onset of neurological deficit were the main reasons for the choice of MRI. Our hospital recently acquired a low-tesla MRI machine, the sixth in a country of more than 90 million people.

It also helped us to rule out associated neurological malformations such as Chiari malformation and hydrocephalus, since the literature reports that spina bifida and its variants are frequently associated with these malformations, in about 85% of cases [1]. Of all myelomeningocele cases, approximately 66% are associated with neurofibromatosis type I or Marfan syndrome, and only 22% are isolated cases. Thoracic myelomeningocele occurs in 1%-5% of all dysraphism cases [5], but to our knowledge, this is the first case reported in our region.

## Conclusion

Anterior thoracic myelomeningocele is a very rare central nervous system malformation, particularly in sub-Saharan Africa. Healthcare professionals, especially those working in resource-limited settings, should be aware of this condition, as its early detection is crucial for appropriate management and may reduce the associated morbidity. Neuroimaging, especially MRI of the spinal cord, should be considered as a key tool in the diagnosis of this spinal malformation, especially in the closed and asymptomatic subtypes.

## Patient consent

The father of the child has given written informed consent for the publication of this report and images.

## References

[1] Ntimbani J, Kelly A, Lekgwara P. (2020). Myelomeningocele - a literature review. Interdisciplin Neurosurg.

[2] Trapp B, de Andrade Lourenção Freddi T, de Oliveira Morais Hans M, Fonseca Teixeira Lemos Calixto I, Fujino E, Alves Rojas LC (2021). A practical approach to diagnosis of spinal dysraphism. Radiographics.

[3] Rab A (2013). Prenatal diagnosis and further clinical characteristics of spina bifida. Asian Pac J Reprod.

[4] Mankahla N, Figaji A. (2014). Occult spinal dysraphism. South Afr Med J.

[5] Ahuka O, Toko R, Omanga F, Tshimpanga B. (2006). Congenital malformations in the North-Eastern Democratic Republic of Congo during civil war. East Afr Med J.

[6] Antony J, Neriamparambil AJ, Ma N. (2020). Case Report of an anterior thoracic myelomeningocele: a multidisciplinary approach to surgical management. World Neurosurg.

[7] Park TS, Cail WS, Maggio WM, Mitchell DC. (1985). Progressive spasticity and scoliosis in children with myelomeningocele. Radiological investigation and surgical treatment. J Neurosurg.

[8] Claude KM, Juvenal KL, Hawkes M. (2012). Applying a knowledge-to-action framework for primary prevention of spina bifida in tropical Africa. Matern Child Nutr.

[9] Williams J, Mai CT, Mulinare J, Isenburg J, Flood TJ, Ethen M (2015). Updated estimates of neural tube defects prevented by mandatory folic Acid fortification - United States, 1995-2011. MMWR Morb Mortal Wkly Rep.

[10] Netto JMB, Bastos AN, Figueiredo AA, Pérez LM. (2009). Spinal dysraphism: a neurosurgical review for the urologist. Rev Urol.

[11] Oner AY, Uzun M, Tokgöz N, Tali ET. (2004). Isolated true anterior thoracic meningocele. AJNR Am J Neuroradiol.

[12] Nathan ST, Puno RM, Paiso JMS, Kawakami N. (2011). A rare case of anterior thoracic myelomeningocele with scoliosis—case report and review of the literature. Spine J.

[13] Copel JA, D’Alton ME, Feltovich H, Gratacós E, Krakow D, Odibo AO (2017).

[14] Sollmann N, Fields AJ, O’Neill C, Nardo L, Majumdar S, Chin CT (2022). Magnetic resonance imaging of the lumbar spine—recommendations for acquisition and image evaluation from the BACPAC Spine Imaging Working Group. Pain Med.

